# An Influence of Oxygen Flow Rate and Spray Distance on the Porosity of HVOF Coating and Its Effects on Corrosion—A Review

**DOI:** 10.3390/ma15186329

**Published:** 2022-09-12

**Authors:** Ali Raza, Faiz Ahmad, Thar M. Badri, M. R. Raza, Khurshid Malik

**Affiliations:** 1 Department of Mechanical Engineering, Universiti Teknologi PETRONAS, Seri Iskandar 32610, Perak, Malaysia or; 2 Department of Mechanical Engineering, CUI Sahiwal, Sahiwal 57000, Pakistan

**Keywords:** thermal spray coating, high-velocity oxyfuel, hot corrosion sulfidation, oxidation

## Abstract

Thermal spray coating, exceptionally high-velocity oxyfuel (HVOF), improves the corrosion resistance and wear of metal. Coating parameters play a vital role in the properties of the coating. The quality of coating can be increased by selecting appropriate coating parameters. In the case of HVOF, the oxygen flow rate and spray distance are the most significant parameters that directly influence the porosity and corrosion resistance of the coating. Porosity is essential in thermal barrier coatings for low thermal conductivity, but there is a limit of porosity beyond which it can cause failure. Hence, understanding the effects of these parameters is essential to evaluate and further minimize the porosity in order to improve the corrosion resistance and durability of the thermal barrier coating. This article reviews hot corrosion in thermal barrier coatings, the stages of corrosion, the importance of spray parameters, and the effect of the oxygen flow rate and spray distance on the corrosion resistance of HVOF-sprayed coatings. Afterwards, the coating materials, the substrate, the flow rate of oxygen, the spray distance, and the fuel used during the HVOF spraying process from recent articles are summarized. In summary, this review compares the flow rate of oxygen and the spray distance with the corrosion capacity of the coating under different corrosive environments and materials to optimize these parameters for high-quality coating, which would sustain under high temperatures for future applications.

## 1. Introduction

**Figure 1:**
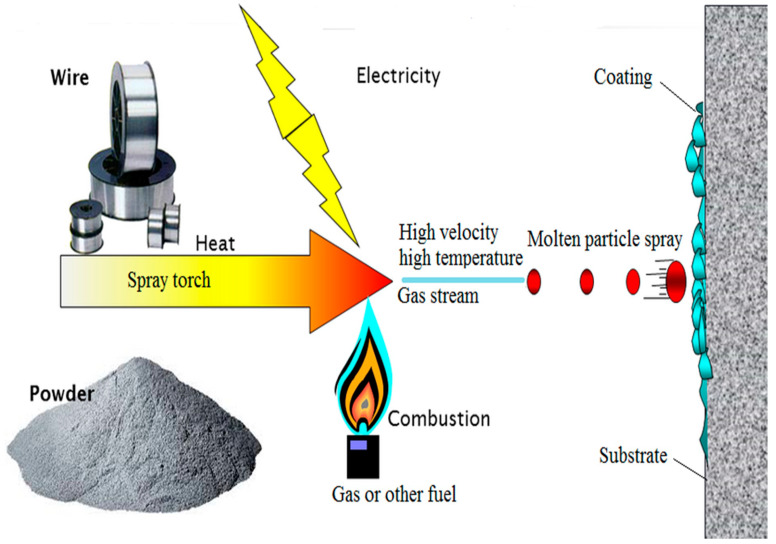
Thermal spray process. Modified with permission from [6]. 2007, Sudhangshu Bose.

Thermal spray is a coating process by which the fine particles of a material in metal or non-metal form are deposited on the surface of a formulated substrate in a molten or semi-molten state [1]. The material to be coated can be deposited in various states, particularly wire, powders, rods, suspension, or molten form [2]. The torch converts particles depending on the energy supplied into a hot gas stream. For combustion, chemical energy is used, and electric power is used for plasma generation [3]. The coating material heated during combustion or plasma generation converts to a molten state and is propelled by the high velocity and high-temperature gas stream to the substrate. The particles distort when depositing the substrate’s surface to produce a splat. When new particles reach the surface, they combine to make multiple splat layers to complete the coating [4]. Figure 1 shows the thermal spray coating process. While selecting a coating material, it should be kept in mind that the thermal expansion coefficient of the substrate and the coating material must match each other [5].

Naturally, thermal barrier coating contains four layers: a ceramic topcoat, a transitional metallic bond coat, a thermally grown oxide (TGO) layer, and a high-temperature base structural material made of superalloy [7]. The topcoat ceramic performs the function of an insulator, keeps the underlying substrate away from degradation due to the high temperature by providing protection, and resists heat transfer [8,9,10,11,12]. The bond coat is used for oxidation resistance [13,14]. The excessively frequently used materials for the topcoat and bond coat are the materials that are heat resistant, such as yttria-stabilized zirconia (8YSZ) and MCrAlY (M: cobalt, nickel, or a combination of both elements), respectively [8]. Thermal spray techniques deposit the topcoat and bond coat on the metallic substrate [15]. The thermally grown oxide (TGO) layer is a barrier against oxygen diffusion [16,17]. A substrate is usually made of cobalt or nickel superalloy to tolerate the load mechanically [18]. During service, it must be kept in mind that the composition and microstructure of the layers change instantly, leading to the failure and degradation of the coating [19].

The life and performance of service materials have improved with the development and research of new thermal coatings [20,21,22,23,24,25,26,27]. Thakare et al. [18] have presented an overview of thermal spray coating materials and technology, the progression in deposition techniques, and the materials used in thermal spray coating. They also introduced the TBC system’s residual stresses, properties, high-temperature performance, and life prediction models. Wu et al. [28] summarized the preparation procedures and improvements in new ceramic materials as well as current problems and future trends. Mehboob et al. [7] reviewed the failure mechanisms of TBCs and methods to alleviate these failures. Gopi et al. [29] addressed the corrosion analysis, wear characteristics, hardness, microstructure, and surface roughness of tungsten carbide coatings sprayed by high-velocity oxyfuel coating. Gupta N. et al. [30] reviewed the mechanical and microstructural properties of the CrC coatings coated by HVOF and their suitability for the applications of piston rings. Vats et al. [31] investigated the outcome of deposition process parameters on the mechanical, wear, physical, corrosion, and erosion properties of coatings deposited by HVOF. Kumar et al. [32] presented an analysis of nanocomposite coatings sprayed by HVOF and concluded that nanocomposite coatings sprayed by HVOF can alleviate the erosion of traditional coatings.

None of the published articles reviewed the relationship between hot corrosion, the oxygen flowrate, and the spray distance based on the literature. This review article presents an outline regarding the high-velocity oxy-fuel technique and hot corrosion in thermal barrier coatings. A detailed discussion is given about the importance of process parameters, the effect of the oxygen flow rate and spray distance on coating porosity, and its effect on corrosion resistance in the case of HVOF coatings. Finally, these parameters were analyzed to recognize the connection between process parameters and the corrosion resistance of HVOF coating for future applications.

## 2. Hot Corrosion in Gas Turbine Blades

Due to combustion gases in gas turbines, the environment is not clean [33]. There are impurities in the fuel or combustion air, such as sea water aerosol containing vanadium and sulfates, deposited on the surface of turbine blades. Hot corrosion is due to the severe deterioration of metals due to sulfidation or oxidation reactions of the deposits in the form of liquid or semi-liquid at an operating temperature. Corrosion has a destructive impact on the structure of components [34]. Corrosion can be prevented by using protective measurements [35].

**Figure 2:**
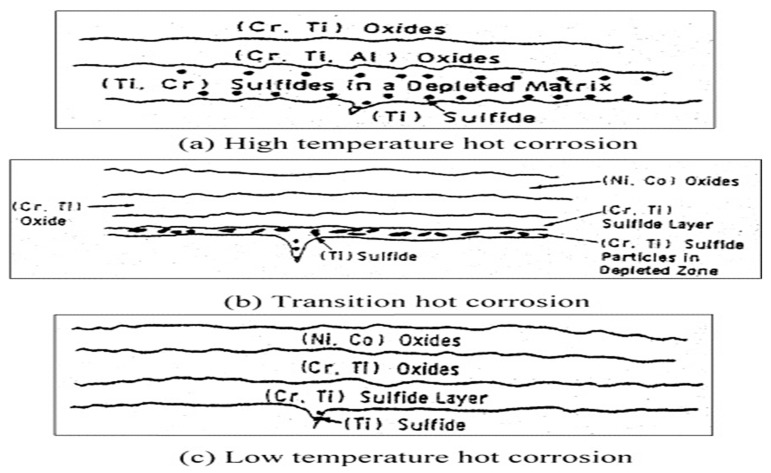
Hot corrosion mechanism in a gas turbine blade. Reprinted with permission from [42]. 2004, M.R Khajavi, M. H Shariat.

In the case of gas turbines, the temperature can reach 1500 °C. Typically, the material used for coating is made of YSZ, which is stable up to 1200 °C. Above this temperature, the coating starts to damage. Due to this, the turbine’s lifetime is decreased at high temperatures. The corrosion of superalloys or materials in the presence of oxidizing gas due to molten salt at a high temperature of 700–925 °C is called hot corrosion. The oxidation rate is fast at this temperature when alloys and metals are contaminated with salts. As a result, the protective oxide layer breaks down, and salts reach the metal surface, causing degradation [36]. Due to this degradation, the bond coat and topcoat are affected because of the porosity of the APS method in YSZ coating. Through pores and cracks, the molten salt penetrates the YSZ and reacts with a bond coat. [37] It is different from corrosion at low temperatures. Suppose the hot corrosion occurs at a temperature equal to the melting point of salt layers. In that case, it is hot corrosion of type 1, and the corrosion occurring at a temperature less than the melting point of the salt layers is called type 2 hot corrosion. The temperature range for high-temperature corrosion is 800 to 900 °C, and for low temperatures, it is 700 to 800 °C. Figure 2 represents the hot corrosion mechanism at low and high temperatures of a substrate.

In a gas turbine, there are corrosive particles in the environment and lower-grade fuel—for example, chlorine, sodium, sulfur, and vanadium [38]. Corrosion in the topcoat is because of lead (Pb) and vanadium in the fuel. Vanadium forms vanadium pentoxide by reacting with oxygen. Sulfur is also present in the fuel that reacts with NaCl and oxygen in the air intake to produce Na_2_SO_4_. These two products are hazardous for the topcoat [39]. These species have pits and voids at the grain boundary responsible for the flow of corrosive particles that react with the metal to deteriorate them, and in this way, the life of the components is decreased. Corrosion in the bond coat can be produced due to chlorides and sulfates of sulfur or potassium gas. In the air, potassium or sodium chloride reacts with sulfur and oxygen in the fuel to have potassium or sodium sulfates. These sulfates are the reason for corrosion in the bond coat. Despite the fuel or air, corrosion can also be produced after combustion due to the contaminants in the fuel that deposit on the surface. After that, these corrosive products stick on the surface of hot sections such as turbine blades. A non-defensive porous oxide scale is produced on the surface, and the material is consumed quickly. As a result, the ability of a material to carry a load is diminished, which is the reason for the failure of the components.

On the other hand, molten deposits are also responsible for the hot corrosion of thermal barrier coatings. The first mode of corrosion by molten deposits is chemical reaction attack by-products—for example, vanadium and sulfur. These products react with ceramic oxides to form corrosive melts. The second mode is mineralization due to the phase reaction of nonreactive liquid, which moves the nonequilibrium phase to the equilibrium phase. The third mode is the corrosion of the bond coat, in which corrodent melts or corrosive gases can enter the zirconia layer under some conditions and corrode the bond coat, eventually leading to the failure of TBC. The fourth mode is the physical damage of coating by the penetration of molten salt through the grains of TBC [40]. So, there is a need to keep the hot segment parts away from molten salt particles at high temperatures. A potentiodynamic polarization test in the saline solution measures the corrosion resistance. The current density for corrosion is between the range of 10^−5^ to 10^−6^ A/cm^2^ [41]. If the turbine blade is not coated, then pitting and corrosion can affect the blade, and the consequences may be severe if there is a salty environment.

There are two stages of hot corrosion in coatings and alloys. These are the initiation stage and the propagation stage. The initiation stage is also called the incubation period. The protective scale is produced at the start of this stage, which variates with time. Each metal may grow its composition and structure of the scale in this period, so it is considered a clean metal surface in an actual gas turbine. Usually, the superalloys that produce protective scales are alumina formers and chromia formers. The length of the initiation stage is affected by many parameters—for example, the fabrication condition, alloy composition, gas velocity and composition, specimen geometry, erosion, temperature cycling and temperature, salt deposition rate, and composition and condition of salt. The protective scale may break down due to some mechanisms. One is basic and acidic fluxing of the scale or a portion of it. Other mechanisms to be considered are the formation of sulfide or growth stress under the scale, thermal cycling due to mechanical cracking of the scale, and modification of the scale due to chloride. The duration of the first stage depends on several factors—for instance, the fuel-air ratio, the content of chlorides in the fuel impurities and the S content of the fuel, the intake air, the level of contaminant of the fuel, the composition, the quantity of deposit, the difference in temperature between the flue gas and the surface of metal, and the composition and temperature of the material.

The same parameters are responsible for the corrosion rate as for the initiation stage for the propagation stage. Corrosion resistance can be improved by increasing the chromium content, which helps extend the incubation period and reduces the corrosion rate in the propagation stage. However, the W and Mo contents can destroy the corrosion resistance property of chromium if they are about 5% in value by supporting the acidic fluxing of alloy. The corrosion rate is affected by heat flux. If the blades are cooled, there is a chance of an increased deposition rate, and a shift into a region with acidic fluxing of low temperatures with high corrosion rates should be avoided. Internal sulfidation due to the transport of sulfur crossways to the protective scale is the reason for the cracking of the scale because of produced stresses in the scale [43].

## 3. High-Velocity Oxy-Fuel

**Figure 3:**
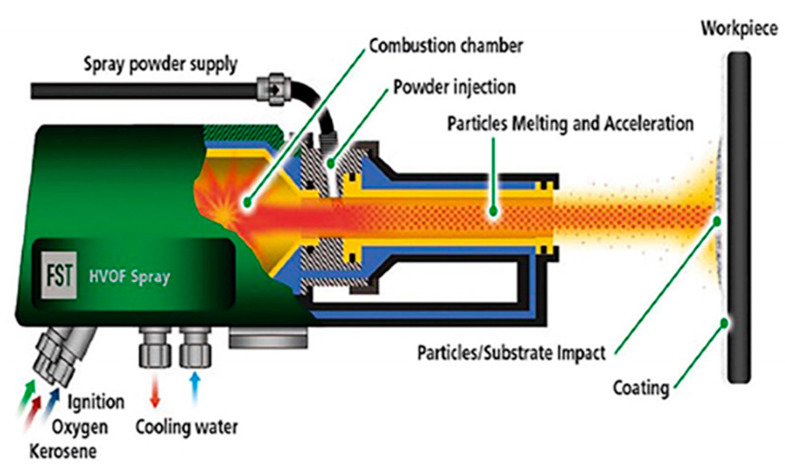
HVOF working principle. Reprinted with permission from [45]. 2021, G. Padmavathi.

High-velocity oxy-fuel (HVOF) coating is a thermal spray technique by which liquid or gas fuel (acetylene, propane, kerosene, hydrogen, propylene) and oxygen are mixed and burned in a combustion chamber. As a result, gas in the form of a stream is produced with a high pressure and temperature, passing through a converging/diverging nozzle to increase the speed at a supersonic level of about 1000 m/s. The temperature induced in this process can be 2500 to 3100 °C, depending on the oxygen and fuel ratio [44]. Alloys in the form of powder are injected into this stream which melt and force it towards the coating surface at a high velocity. Excellent bonding and dense and thick coatings with a low number of oxides are produced by this technique due to inert shrouding and high particle velocities. Due to the high speeds of particles, their flight time is reduced, and the oxidation reaction has less time to occur. Thus, coatings deposited through the HVOF technique have less oxidation. Applying thermal and kinetic energy to the particles enables powerful bonding on the surface of a substrate. Figure 3 shows the schematic of the HVOF technique. In this technique, continuous fuel burning is achieved because the porosity is very low, and dense coating is obtained with a high corrosion resistance. Coatings through this technique are tough and have a good resistance to abrasion, heat, and wear. Thermal spray coating, mainly HVOF, improves surface corrosion resistance and wear [45]. The HVOF process is applied in numerous industrial applications to protect components from corrosion [44,46]. Many authors have stated the benefits of this technique’s high corrosion properties for producing dense protective coatings.

After selecting the proper technique for coating, it is imperative to optimize the coating parameters. Coating parameters have a significant function in the properties of the coating. The quality of coating can be increased by selecting appropriate parameters. Every technique has its coating process parameters. In the case of HVOF, these parameters are the oxygen flowrate, the flowrate of liquid or gas fuel (acetylene, propane, kerosene, hydrogen, propylene), the distance between the substrate and the spray nozzle, the coating powder flowrate, the powder shape, the particle size, the particle velocity, the particle temperature, the spray angle, and the substrate preheating. The process parameters are independent variables, while their outcome consists of dependent variables, including thickness, porosity, hardness, toughness, wear, corrosion, and oxidation resistance. Many statistical tools are used to optimize the parameters. One of them is the Taguchi method, which is a very effective and powerful method to improve the process parameters, as reported by many researchers [47,48].

Much work has been done to optimize the parameters of HVOF. For example, Vignesh et al. [49] tried to achieve minimum porosity and maximum hardness by optimizing the spray parameters of HVOF. The parameters were the fuel and oxygen flowrate, the powder feed rate, the spray distance, and the carrier gas flowrate. Among them, the oxygen flowrate appeared to be the compelling factor influencing the porosity and hardness of the coating. Porosity depends on the thermal energy and kinetic energy of the particles. Less porous coatings can be obtained with less thermal energy and high kinetic energy. Jet velocity and flame temperature can be controlled by selecting the appropriate oxygen to fuel ratio. The amount of free oxygen is low in low temperatures but increases in high flame temperatures. Due to this increased amount, the flame becomes heavier and has less of an ability to carry the powder particles, leading to low kinetic energy. Therefore, it is a central part of coating to control the O/F ratio. Wang et al. [50] studied the iron-based amorphous solid and its processing parameters’ outcome. It was observed that the fraction of un-melted particles and porosity increased with the powder feed rate and decreased with the ratio of oxygen/fuel. Using different spray parameters, three metallic glass coatings based on Fe were prepared by Zhang et al. [51]. It was found that the porosity decreased by increasing the spray powder feed, while the amorphous phase content first rises and then declines.

A study [41] reported the effect of the processing parameters for tungsten carbide coating on a nickel substrate. It was observed that the hardness, compressive residual stress, corrosion resistance, wear, and toughness were improved by selecting appropriate parameters. The deposition process strongly affects the performance of coatings based on WC. The structure and composition of the feedstock powder are also important [52]. Li Chang et al. [53] modelled the flow field of the HVOF process using kerosene as a fuel. Velocity, temperature, and trajectory were simulated for WC-12Co particles. They studied the effect of the oxygen/fuel ratio, particle shape, size, and injection angle on the flame flow. It was noted that the oxygen/fuel ratio directly affects the characteristics of flame flow. They calculated that a particle size of 10–30 µm and an injection angle of a particle of 45° is the best to obtain a high-quality coating. Zois, D et al. [54] sprayed stainless steel powder using the HVOF technique and optimized the spray parameters statically. They stated that corrosion resistance is mainly affected by the porosity of coating, and porosity is primarily affected by the oxygen to fuel ratio. Coating porosity is also reduced by short and moderate spray distances.

## 4. Effect of Oxygen Flowrate on Corrosion Resistance

For WC-CoCr coatings, the outcome of the oxygen-to-fuel ratio on the corrosion resistance was reported by Picas et al. [55]. It was noted that porosity reduces by increasing the oxygen-to-fuel ratio, and a decrease in porosity is the reason for improved corrosion resistance. Huang et al. [52] fabricated the nanostructured WC-10Co4Cr coatings by the HVOF technique and investigated the coatings’ mechanical, microstructure, erosion, and corrosion resistance by changing the process parameters. The oxygen flow rate used in the experiment was 100 L/min, and the coating thickness was around 250 µm. They observed that liquid fuel thermal spray has a higher corrosion resistance than gas-fueled spray coating. The corrosion resistance also depends on the crystalline phase of the coating. In a study [56], the effect of the temperature of WC-Co particles on the corrosion resistance and porosity was studied. The spraying parameters were the oxygen/fuel ratios of 2.5, 3.5, and 3.9 and the particle temperatures of 1702, 1880, and 2012 °C, respectively. It was observed that the corrosion resistance depends on two factors: porosity and nanocrystalline amorphous phases. The highest corrosion resistance was achieved with the sample with a porosity of 1.7% and a particle temperature of 2012 °C. These factors increase by increasing the temperature during coating and the oxidation of the surface. Corrosion resistance decreases by increasing the first factor and increases by increasing the second factor. However, oxidation’s harmful effect on the porosity should be considered to achieve a high corrosion resistance. Corrosion depends on the dominant factor. The ability to control the fuel and oxygen flow and the combustion chamber parameters by using an HVOF system is essential. The high temperature of the particles is a critical parameter in this case.

Qiao Li et al. [48] studied the influence of HVOF spray parameters on the corrosion resistance of iron-based alloy powder coating on 45 steel substrates. They used the Taguchi method to learn the spray distance and fuel flow outcome on the coating performance. The corrosion performance of the coating was examined by applying an electrochemical workstation. The best oxygen flowrate was 1840 and 2000 scfh and showed a dense structure with a porosity of nearly 1%. The coating with a lower porosity displayed an improved resistance against corrosion. Huang Yan et al. [52] reported the corrosion behavior of HVOF-sprayed WC-CoCr coatings in an NaCl solution. The oxygen flow rate was 100 L/min, and the substrate was 304 stainless steel. The coating thickness was about 450 µm. They observed that the HVOLF WC-10Co4Cr coating showed the best electrochemical and mechanical properties compared to those of the HVOFG. Berger et al. [41] investigated the corrosion property of coating on Superduplex alloy using HVOF. They used three coating powders and process parameters: oxygen flow 0.875 m^3^/s and standoff distance 330 mm. The corrosion resistance was evaluated with the help of polarization curves in a salty environment of NaCl using galvanostat. The best results were obtained for a coating thickness of 135 µm and a porosity of 3.7%, with a 0.0002 mm/year corrosion rate.

The result of the grinding depth of cut on the corrosion behavior of WC-10Co-4Cr coating by the HVOF process was investigated by Pishva et al. [46]. The substrate used was a carbon steel disk, the oxygen flow rate was 835 SL/min, and the coating thickness was 400 µm. The corrosion behavior of the coated samples was evaluated in a 3.5 wt.% NaCl solution using Ivium Potentiostat (Netherlands), and the samples were tested by the open circuit potential, electrochemical impedance spectroscopy, and the potentiodynamic polarization curve. By increasing the depth of the grinding cut, the porosity increased, and the coating resistance against corrosion decreased. Mardali et al. [57] investigated the effect of MgO on hydroxyapatite coating by HVOF. The oxygen flow rate was 850 L/min, and the coating thickness was 11.8 µm. Electrochemical impedance spectroscopy was used to analyze the corrosion resistance of coating in a simulated body fluid using a computer-controlled potentiostat system. The corrosion resistance of the coating increased due to the presence of the MgO layer. Veronica et al. [58] deposited alternative hard metals—WC-NiMoCrFeCo, WC-FeNiCrMoCu, WC-FeCrAl, and WC-CoCr—using HVOF on a stainless-steel substrate. The oxygen flowrate for fine WC-CoCr was 214 SLPM less than that for medium WC-CoCr powder, with a flowrate of 234 SLPM to prevent unnecessary decarburization. For WC-FeNiCrMoCu powder, the gas flowrate was lower than that for WC-NiMoCrFeCo powder because of the fine size of the WC primary particles, and Fe-based alloys are more disposed to oxidation than Co or Ni alloys. The range of thickness for the coating was 200–300 µm. The corrosion test was done in a 3.5% (*w*/*v*) NaCl aqueous solution. The corrosion resistance of WC-NiMoCrFeCo was better than those of the other coatings, having the lowest corrosion current density with an oxygen flowrate of 228 SLPM.

Murariu A.C. et al. [59] studied the effect of the saline environment on the fatigue behavior of WC-CrC-Ni coating sprayed by HVOF on carbon steel bars. The saline environment was prepared for corrosion test by mixing 3% NaCl in water. Using an oxygen flow rate of 200–220 L/min, the best results were obtained for a corrosion thickness of 110–220 µm. Due to the corrosion environment, the fatigue life of the specimen was reduced by 30%. Madu G. et al. [60] evaluated the hot corrosion behavior of Cr_3_C_2_-35NiCr coating sprayed by HVOF on an SS 304 steel substrate. The oxygen flow rate was 270 LPM. A simulated boiler environment was used for hot corrosion testing at 800 °C for 50 cycles. The coated sample exhibited a better hot corrosion resistance than the uncoated substrate. Pala Zdenek et al. [61] reported the corrosion behavior of the Stellite 6 alloy on a 304 stainless steel substrate using the liquid fuel-based HVOF technique. The oxygen flow rate was 920 LPM, and the coating thickness was 250 µm. For the corrosion test at high temperatures, the samples were left bare or deposited in KCL prepared in ethanol and placed in alumina crucibles under a controlled environment in a tube furnace at 700 °C for 250 h. To avoid corrosion at low temperatures, the samples were heated and cooled by flowing nitrogen. After exposure, there was a change in the phase transformation and microstructure within nearly 15 µm of depth in the bare samples. In the case of KCL-deposited samples, severe corrosion was observed in terms of a chromium depletion of almost 280 µm.

Vignesh S. et al. [62] studied the electrochemical corrosion performance in an NaCl solution of iron-based metallic amorphous coating on an AISI 316 stainless steel substrate sprayed by HVOF. The oxygen flow rate was 252 L/min, and the coating thickness was 300–350 µm. The corrosion resistance was evaluated using the potentiodynamic polarization test according to standard ASTM G3-89. The OCP value of the coating was more significant than that of the substrate, indicating a more excellent corrosion resistance. Boleli G. et al. [63] produced two TiC-FeCrAl coatings on a stainless-steel plate using HVOF. For set 1, the oxygen flowrate and thickness were 214 SLPM and 307 µm; for set 2, the oxygen flow rate and thickness were 188 SLPM and 520 µm, respectively. In the case of WC-CoCr, the oxygen flow rate was 214 SLPM. The corrosion resistance was determined using an electrochemical polarisation test in 0.1 M HCL solution at room temperature. The corrosion resistance of WC-CoCr was better than that of other coatings. Ding Xiang et al. [64] deposited WC-10Co4Cr coating on 304 stainless steel specimens by two HVOF systems. The oxygen flux was 56.6 m^3^/h for HVOLF and 100 L/min for HVOGF, and the thickness of the coating was around 450 µm. Before impacting the substrate, the velocity and temperature of the coating droplets were 835 m/s and 1850 °C for HVOLF and 650 m/s and 1910 °C in the case of HVOGF, respectively. The CorrTest electrochemical test measured the electrochemical performance for 30 min. The current corrosion density and potentiodynamic potential were obtained from the potentiodynamic polarization curve using the CView software. The coating prepared by HVOLF had a lesser corrosion current density and more potentiodynamic potential than the other coatings, indicating a better corrosion resistance. This better electrochemical property can be traced back to the dense microstructure having a porosity of 0.31%, less than 1.28% of the other coatings.

Amudha A. et al. [65] studied the corrosion performance of NiCrs-CrC2 coating on a low carbon steel substrate by the HVOF technique. The particle size of the coating powder was −45/+15 micron, and the oxygen flow rate was 200 L/min. The electrochemical system studied the corrosion resistance of the coating according to the ASTM G61 standard. The corrosion rates were assessed by EIS and LPR techniques in a 3.5% NaCl solution with a 10 KHz–100 mHz frequency range. The corrosion current density of the substrate was five times greater than that of the coating, which implies the better corrosion resistance of the coated sample. Yao Sun et al. [66] specified the corrosion behavior of WC coatings with three binders—WC-12Ni, WC-10Co4Cr, and WC-12Co—on a JIS S31C steel substrate. The substrate was preheated to 100 °C before spraying with an HVOF system. The oxygen flow rate was 42 L/min, and the coating thickness was 200 µm. A salt spray test was used according to the standard ASTM B117-03, and 50 g of NaCl with one liter of deionized water was used as a salt solution to evaluate the corrosive behavior. The test duration was 31 days, and the ASTM B537-70 standard was used to assess the test surface. The corrosive performance of WC-10Co4Cr was better than that of the other two coatings.

Sassatelli et al. [67] reported in their findings that the corrosion resistance of coatings based on Co alloys is potentially influenced by the microstructure of the coating and the process parameters. They used gas atomized Co-based powder for coating and AISI 304 stainless steel as a substrate. The oxygen flow rate varied between 188–214 SLPM for six runs, and the oxygen pressure was 1.172 MPa. The coatings sprayed with high temperatures close to the stoichiometric ratio appeared denser than the others, with a porosity of less than 1% and thicknesses of 491, 665, 701, and 885 µm. For corrosion, an electrochemical polarization test was used for each set of process parameters, with 300 mL of 0.1 M HCl as an aqueous solution.

Additionally, the samples with the lowest porosity were subjected to the Corrodkote test according to standard ASTM B380-97 (2013) for 100 h (five cycles). It was concluded that coatings with less than 1% porosity effectively protected the substrate. The microstructure of the coating influences this corrosion resistance, while the process parameters influence the tribological properties less. Murariu Alin et al. [68] presented the performance of WC-CoC-Ni coating powder coated on stainless steel and carbon steel by HVOF. The oxygen flow rate was 185 L/min, and the coating thickness varied between 114 and 647 µm. Assessment electrochemical methods were used for corrosion resistance. For a thickness of 110 to 220 µm, the best experimental results were obtained for corrosion resistance compared to those for a thickness of 400–647 µm. In addition, the corrosion resistance rate increased 1.5 times for the samples that were surface processed (polishing) after the deposition of the coating.

Farokhian Gh et al. [69] examined the HVOF coating of WC–20Co–1Ni powder over the lifetime of a downhole drilling motor constructed of stainless steel. The flow rate of oxygen and the coating thickness were 761 L/min and 88.82 µm. Then, 0.1 N HCl solution was used to compare the corrosion potential of hard chromium plating and WC–20Co–1Ni coating. According to the electrochemical results, the tendency of oxidation and corrosion of the WC–20Co–1Ni coating was minor compared to that of the hard chromium plating. Sarkar Kuntul et al. [70] prepared composite coating with blast furnace pig iron on a mild steel substrate using HVOF. The oxygen flow rate and coating thicknesses were 240 SLPM and 19–37 µm, respectively. An electrochemical study was conducted using electrochemical impedance spectroscopy, potentiodynamic polarization, and linear polarization. According to the results, the coating showed a high corrosion resistance comparable with SS coating and expiatory effects to mild steel in a 3.5% NaCl freely aerated solution. Wei Zheng et al. [71] fabricated the (AlCoCrFeNi)1-X (WC-10Co) X composite coating on 6Cr13Ni5Mo steel by HVOF. The Taguchi method was used to study the effects of spray parameters. The optimized oxygen flow rate was 802.2 LPM. An electrochemical test was conducted in a 3.5 wt.% NaCl solution with a three-electrode system. The corrosion of the coatings was better than that of the steel substrate.

Zouari S et al. [72] proposed a study of two coating powders, 316L stainless steel and (NiCrBSi) alloy, on the brass substrate using HVOF. The oxygen flow rate was 180 NL/min, and the coating thickness was 158.5 and 195 µm. The salt fog method was used to carry out a corrosion test. The samples were suspended on frame in an acetic acid salt spray for 96 h. Compared to 316L SS, the corrosion resistance of the NiCrBSi coating was high, and no substantial defects were observed. It was concluded that better coating quality might be developed by optimizing spray parameters. Wei Zheng et al. [73] fabricated the AlCoCrFeNi coating alloy using HVOF deposited on 06Cr13Ni5Mo stainless steel. The flow rate of oxygen was 802.2 L/min. The average porosity and thickness of the coating were 0.36% and 250 µm, respectively. Electrochemical impedance spectroscopy and a cyclic potentiodynamic polarization test were conducted in a 3.5% NaCl solution. The corrosion resistance of steel was higher than that of the high-energy alloy coating. Galvanic corrosion was the primary corrosion type of coating. Li Deyan et al. [74] evaluated the outcome of the amorphicity of Fe_53_Cr_19_Zr_7_Mo_2_C_18_Si coating coated on 316L stainless steel by HVOF on corrosion performance. The coating thickness was 150 µm, and the oxygen flow rate was 800 mL/min. Next, the sample was annealed at 750 °C for 1 h and cooled naturally to room temperature in the furnace. Potentiodynamic polarization testing was done to examine the electrochemical behavior of the coating. It was noted that the corrosion resistance of the as-sprayed coating was higher than that of the heat-treated coating due to fewer structural defects and chemical homogeneity. At the same time, the anti-wear performance of the annealed coating was better than that of the as-sprayed coating.

Pukasiewicz et al. [75] presented the corrosion resistance of FeMnCrSiNiB alloy coating deposited on an SAE 1020 substrate by the HVOF technique. The oxygen flow rate and pressure were 606 SCHF and 150 psi, respectively. A method of potentiodynamic polarization was used to determine the corrosion rate and potential. According to the results, the corrosion rate of the coating was 4.36 × 10^−7^—lower than that of carbon steel, with a value of 42.30 × 10^−7^, but higher than that of stainless steel, with a value of 1.05 × 10^−9^. Aoudia K et al. [76] evaluated the outcome of surface mechanical attrition treatment (SMAT) on the corrosion resistance and mechanical properties of NiCrBSi coating powder, with an average particle size of 45 µm deposited by HVOF. E355 steel was sued as a substrate, and the thickness of the coating was around 400 µm. The flow rates of oxygen and kerosene were 897 L/h and 22.5 L/h, respectively. SMAT is a surface modification process based on the vibration and movement of balls which is used to enhance the corrosion resistance and wear of many components. It was carried out by placing balls in the cylindrical chamber and by agitating via an ultrasonic generator to produce vibrations. After coating, the SMAT was performed, which resulted in a more compact surface with no porosity. Potentiodynamic polarization curves were used to evaluate the electrochemical behavior using ModuLab potentiostat. The polarization resistance and open circuit potential were assessed for 72 h. It was noted that the coating corrosion resistance improved by SMAT compared to that for untreated coating.

Alnaser Ibrahim et al. [77] focused on the high-temperature corrosion behavior of Ni-based superalloy coated through HVOF and APS with Cr_3_C_2_-25NiCr powder at 950 °C under molten salt and an air oxidation environment. HVOF coating was performed using jet fuel and oxygen, with flow rates of 20 and 840 lpm. A high-temperature corrosion test was performed for 50 cycles on deposited and undeposited samples. These coatings can withstand 50 corrosion cycles without stripping the base metal. Li Yu Wan et al. [78] prepared WC-12%Cr_3_C_2_-6%Ni coating on 42CrMo steel by HVOF to observe the corrosion behavior of coating before and after aluminum phosphate sealing. During spraying, the oxygen flow rate and pressure were 17 L/min and 0.56 MPa. An immersion corrosion test was carried out with a beaker and water bath in 3.5% Na_2_SO_4_ according to the ASTM G31-72 (2012) standard. The test time and temperature were 28 days and 35 °C. The weight-loss method was used to test the corrosion resistance of the coating. Using aluminum phosphate sealing treatment, the porosity of the coating decreased to lower than 2%, due to which no apparent coating/substrate interface corrosion occurred.

Verdian M.M. [79] investigated the corrosion behavior of Stellite 6 on an ASTM A283 steel substrate using HVOF. A corrosion test was performed in an NaCl solution at 25 °C. The oxygen and kerosene flow rates for the spray process were 260 mL/min and 83 L/min. Based on the results, the corrosion rate of the steel substrate was higher by orders of magnitude than that of the stellite coating. The work done by previous researchers on HVOF coating using different flow rates, fuels, substrates, and coating materials is in Table 1. The oxygen flow rate depends on the fuel and spray gun used during the coating process. In the case of kerosene fuel, the oxygen flow rate is 500–900 L/min. For LPG and propane, the flow rates are 250 L/min and 100–250 L/min, respectively. Likewise, the gas flow rate depends on the coating material used. The gas flow rate would be comparatively low for a material if the size of the particles is acceptable or if it is more disposed to oxidation than other alloys [58].

## 5. Effect of Spray Distance on Corrosion Resistance

The spray distance affects the phase degradation, deposition efficiency, porosity, and bonding strength. The thermal influence and splash rate in a lower kinetic energy and a small distance have an inverse effect on the porosity. Azizpour et al. [56] inspected the impact of spray distance on the corrosion resistance of WC-Co coating. They used three samples with different spray distances of 170, 200, and 250 mm. They observed that a sample with a spray distance of 170 mm has the highest corrosion resistance. Qiao Li et al. [48] studied the impact of HVOF spray parameters on the corrosion resistance of iron-based alloy powder coating on a 45 steel substrate. They used the Taguchi method to learn the spray distance and fuel flow outcome on the coating performance. The corrosion behavior of the coating was studied by applying an electrochemical workstation. They confirmed that a spray distance of 380 mm was optimal for coating and showed a dense structure with a porosity of nearly 1%. The coating with a lower porosity displayed an improved resistance against corrosion. Vasudev et al. [44] reported the electrochemical corrosion behavior of Inconel 718 coating on the gray cast iron substrate using HVOF. The standoff distance was 205 mm for the topcoat, 200 mm for the bond coat, and 50–60 µm for the thick bond coat. To study the corrosion behavior of the bare and coated samples, an electrochemical corrosion test was used in which potentiodynamic polarization Tafel curves were drawn. The specimen was exposed to 3.5 wt.% NaCl solution at ambient temperature for 1 h. An SEM micrograph was used to analyze the kinetics of corrosion. There was a substantial decrease in the corrosion rate of the coated samples compared to that of the bare samples. This performance was affected by the porosity content, which was less than 1%.

**Figure 4:**
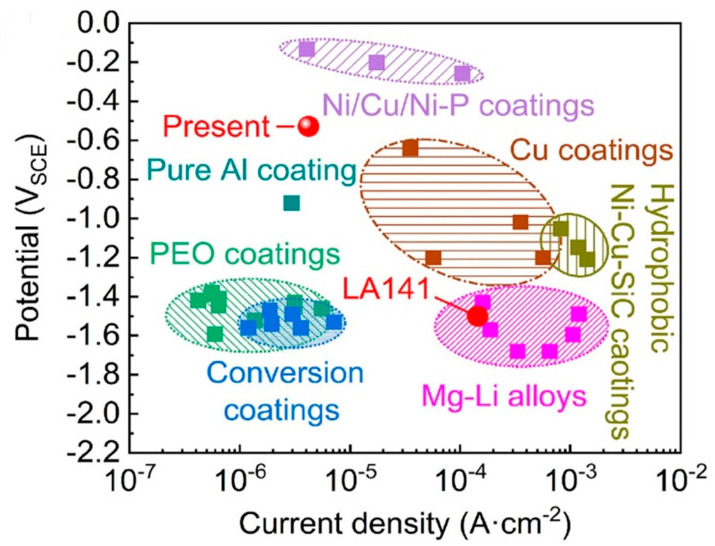
Corrosion potential and corrosion current density of Mg_Li alloys. Reprinted with permission from [105]. 2021, Y. J Sun.

Anushri Nag et al. [104] explored the performance of Cr_3_C_2_25Ni and WC-12 Co coatings in the molten zinc deposited by the HVOF method on an SS 316L substrate. The spray distance was 175 mm, and the coating thickness was 300 µm. To check the corrosion resistance, the coated samples were dipped in a molten zinc bath for 30 days, and the temperature was kept at 455 °C. To understand the deprivation mechanism of coating about corrosion phases, Raman spectroscopy was performed. The coating degraded significantly; however, the alumina coating remained inert to the molten zinc. Sun Y.J. et al. [105] reported the properties of Fe-based amorphous coatings on a magnesium alloy substrate by HVOF. The spray distance was 350 mm, and the thickness varied from 150 to 300 µm. Potentiodynamic polarization curves determined the corrosion resistance. According to the corrosion potential, the corrosion resistance of the Fe-based coating was lower than that of the Ni60 interlayer due to the high porosity and low amorphous content. Figure 4 shows the corrosion potential and corrosion current density of Mg-Li alloys. Birdeanu A. V. et al. [106] presented the coating characteristics of TiO2 deposited by HVOF using two processing paths of preparing substrates by sandblasting and fast laser textures. Carbon steel electrodes were used as a substrate, and the standoff distance was 200 mm between the nozzle and substrate. The corrosion properties of the two surfaces, one deposited by HVOF and the other first textured with the laser and then deposited by HVOF, were estimated in NaCl. The potentiodynamic polarization electrochemical method with a representation of Tafel and the open circuit potential measurement (OCP) was used to investigate the corrosion protection. The laser-textured substrate coated with HVOF showed an improved corrosion, IE 66.64%, compared to that of a classical HVOF coating, IE 59.61%.

Zhang Haijun et al. [107] studied the cavitation erosion resistance of Co-based coating, WC-10Co4Cr coating, Co-based/WC-10Co4Cr composite coating, and Fe-based amorphous coating in artificial seawater and deionized water sprayed by HVOF. The coatings were deposited on 316L stainless steel, and the spray distance was 300 mm for all coatings. The coating porosity was measured in the range of 0.6–2.7%, with the Co-based coating as the lowest porosity. To examine fluid cavitation, erosion cavitation tests were completed in vibratory cavitation equipment according to the standard ASTM G 32-16. Corrosion has a harmful impact during the cavitation of materials, and in seawater, cavitation erosion processes are very complex. It is reported that when cavitation happens in corrosive media, electrochemical corrosion and mechanical cavitation coexist, resulting in the acceleration of material deterioration and the volume loss rate. After the tests of artificial seawater and deionized water, the results revealed the best erosion cavitation resistance of WC-10Co4Cr coating in artificial seawater and deionized water. Asl Shahin et al. [108] reported the effect of heat treatment on the corrosion behavior of WC-17Co coating deposited on a mild steel substrate by HVOF. The coating distance was 280 mm. Coated samples were heat-treated in a vacuum in a chamber and heated up to 1100 °C at a rate of 100 °C per minute. An electrochemical corrosion study was performed by conducting a corrosion test and plotting potentiodynamic polarization curves. The results showed that the heat-treated coating decreased adhesion to the substrate, but the as-sprayed coating had a dense structure. The corrosion resistance of WC-17Co coating heat-treated at 1100 °C decreased due to the formation of η phase during the heat-treatment process.

Zhang Haijun et al. [109] fabricated Fe_53_Cr_19_Zr_7_Mo_2_C_18_Si coating using HVOF to study their corrosion resistance behavior for marine applications. Oxygen and kerosene were used as fuel gases, and the spray distance was 300 mm. 316L stainless steel was used as a substrate. After coating, the specimens were heat-treated at 750 °C for 1 h and then cooled naturally to room temperature in a vacuum furnace. A corrosion test was conducted in a NaCl solution for 1 h. It was revealed that the as-sprayed coating exhibited outstanding enhanced resistance against corrosion compared to the heat-treated coated specimens. The corrosion behavior in molten zinc, the mechanical properties, and the abrasive wear of boride coatings deposited by HVOF were pointed out by Chen Xiao et al. [110]. Four pure powders, B, Mo, Cr, and Co; Ni, B, Cr, and Mo; Co, B, Cr, and Ti, were mixed in ball milling to prepare the mixture powders. These powders were deposited on 316L stainless steel material with a standoff distance of 200 mm. A corrosion test was conducted in molten zinc, in which specimens were dipped at a temperature of 460 °C for 360 h. The corrosion resistance of the substrate material increased due to coating, and compared to the TiB/CoCr and MoB/CoCr coatings, the MoB/NiCr coating had the highest corrosion resistance against molten zinc.

The corrosion mechanism of Fe-Cr-B coatings in a simulated biomass combustion environment was studied by Reddy Liam et al. [111]. The coating powder Weartech SHSTM 7574 (B 2.96, Cr 17.78, Mo 14.24 Mn 2.10, W 5.90, C 0.88, Ni 0.13, Si 1.36 and Fe balance wt.%) was deposited on a 304 stainless steel substrate by HVOF. The fuel used in the spray gun was kerosene, and the interpass distance was 4 mm. In a tube furnace, a corrosion test was performed in a controlled environment containing 500 ppm HCl, N_2_ balance, and 5% O_2_ at room temperature at 700 °C. Before corrosion exposure, the thickness of the coating was measured at 235 µm. After the test, the thickness loss of the coating was negligible, and better corrosion resistance compared to that of the laser clad sample was observed due to the high fraction of the amorphous microstructure of the coating. The hot corrosion behavior of conventional and nanostructured Cr_3_C_2_NiCrBSi coating deposited by HVOF was presented by Shankar R. et al. [112]. The coating was carried out on Inconel 718 alloy with a spray distance of 8–10 inches and a thickness of 150–200 µm. A hot corrosion test was conducted on an uncoated and coated specimen in a molten salt environment for 50 cycles. There was heating for one hour in each cycle and cooling at room temperature for 20 min. A muffle furnace was used to heat the specimens at 900 °C. After 50 cycles, the results showed the maximum weight gained by the uncoated substrate, while in the case of nanostructured and conventional coating, the weight gain was 0.4 mg/cm^2^ and 1.8 mg/cm^2^. The nanostructured coating exhibited a better corrosion resistance compared to that of traditional coating.

Mousavi S. E et al. [113] applied Stellite-6 coating powder on a Ni-Al bronze substrate using HVOF to investigate corrosion and wear properties. The distance between the substrate and spray gun was 250 mm, and the particle size of the coating powder was 10–30 µm. A potentiodynamic polarization test was used to evaluate the corrosion behavior of a substrate coated by stellite-6 coating. The test was performed at room temperature in a 3.5% NaCl solution. The polarization curves depicted a low current density compared to the substrate, which decreased from 3.91 to 1.82 µA/cm^2^, showing a high corrosion resistance. Hao Enkang et al. [114] designed the NiCoCrAlYTa/10Ag/5Mo composite coating. They deposited on an Inconel 718 substrate using HVOF to study the influence of molten salt, including V_2_O_5_, on the hot corrosion performance of the composite coating. The mass fraction of powder was 5% Mo, 10% Ag, and 85% NiCoCrAlYTa, and they were mixed by mechanical mixing. In the coating process, the spraying distance was 30 cm, and the coating thickness was measured to be 400 µm. For the hot corrosion test, three samples were prepared in. The first sample was oxidized in the air for 6 h at 800 °C with no salt, and for samples 2 and 3, a hot corrosion test was conducted in two types of salts with a composition of 25 wt.% NaCl, 75 wt.% Na_2_SO_4_ and 20 wt.% NaCl, 75 wt.% Na_2_SO_4_, 5 wt.% V_2_O_5_ respectively. The samples were heated to 800 °C for 6 h and cooled at room temperature in a muffle furnace. It was noted that NaCl-Na_2_SO4-V_2_O_5_ molten salt is extra corrosive compared to NaCl-Na_2_SO_4_ molten salt to the composite coating. The elastic modulus and hardness of the composite coating increased after the hot corrosion test because of the growth of grains and the internal mutual diffusion of elements.

Sreenivasulu V et al. [115] compared the high-temperature corrosion performance of 80 A alloy coated with metallic NiCrMoNb and carbide Cr_3_C_2_-25NiCr powders by using the HVOF process. During the coating process, the standoff distance was 365 mm. The coating porosity and thickness of metallic and carbide coatings measured at 0.54%, 0.67%, 180 µm, and 200 µm, respectively. A high-temperature corrosion test was conducted in a molten salt environment Na_2_SO_4_-60%V_2_O_5_ and in air oxidation up to 50 cycles for both the coated and uncoated samples. During each cycle, the samples were heated at 900 °C for one hour in a tube furnace and then cooled to room temperature for 20 min. The weight change of the substrates was measured after each cycle. After 50 cycles, in the case of the air environment, the weight gain of the coated samples was 1.57 mg/cm^2^, and that of the uncoated substrate was 1.46 mg/cm^2^. After exposure to a molten salt environment, the weight gain of the coated sample was 7.30 mg/cm^2^, and the uncoated substrate was 7.46 mg/cm^2^. Thermogravimetry analysis exposed that metallic coating provided a good resistance against corrosion in the molten salt environment due to less porosity, which can be attributed to the spray distance during the coating process. Ang Andrew et al. [116] developed spraying process parameters to optimize the coating process of WC-10Co-4Cr and Cr_3_-C_2_-25NiCr on a carbon steel substrate. Before coating, the substrate was degreased and preheated, and the standoff distance for coating was 330–380 mm. By reducing the standoff distance from 380 to 330, Cr_3_-C_2_-25NiCr coating was optimized, and the deposition efficiency increased up to 40%. Additionally, the micropores of the coating decreased in the case of a spray distance of 330 mm.

The spray distance with different coating materials and substrates used by previous researchers is illustrated in Table 2. It can be taken into consideration that, for different materials, different spray distances are used. For example, in the case of WC-10Co-4Cr coating, the spray distance is 300–450 mm. The standoff distance for a thermal spray process depends on the spray gun used. Table 3 shows the relation between the oxygen flowrate, porosity, and coating material. It is worth noting that the porosity depends on the oxygen flow rate and spray distance for different coating materials. The porosity is different for the same coating material and has different oxygen flow rates and spray distances. 

## 6. Recommendations and Discussion

There are many process parameters for HVOF coating. Among them, the oxygen flow rate and spray distance are the most critical process parameters that affect the porosity and corrosion resistance of coating. An increasing oxygen-to-fuel ratio decreases the porosity of coating, but less porosity is not always a reason for better corrosion resistance. Other factors influence the behavior of the corrosion resistance of coatings. For instance, the corrosion resistance of different coating materials will differ for the same oxygen flow rate and spray distance.

Similarly, the particle size of coating material is another reason for controlling the corrosion resistance of the coating. A nanostructured coating has a better corrosion resistance than a conventional coating. Overall, many parameters should be kept in mind while selecting a coating material and technique; each one has different applications. However, for the same coating material and technique, the process parameters, including the oxygen flow rate and spray distance, can be optimized to obtain a coating with a high corrosion resistance. Among the above-mentioned parameters, the oxygen flow rate depends on the type of fuel used in a spray gun. From the literature, the primarily used fuels are LPG, hydrogen, kerosene, propane, acetylene, natural gas, and propylene. For kerosene and propane, the range of the oxygen flow rate is about 500–1000 lpm and 100–300 lpm, respectively. The range of the spray distance used by previous researchers is 200–450 mm.

## 7. Conclusions

This review article discusses the effect of the oxygen flowrate and standoff distance upon the porosity and corrosion resistance of the coating deposited by HVOF. The hot corrosion of metals or superalloys occurs due to oxidizing gas with molten salt. The first mode of corrosion by molten deposits is a chemical reaction attack by-product—for example, vanadium and sulfur. The second mode is mineralization due to the phase reaction of nonreactive liquid, which moves the nonequilibrium phase to the equilibrium phase. The third mode is the corrosion of the bond coat, in which the corrodent melts or corrosive gases corrode the bond coat.

(1) Corrosion resistance can be improved by increasing the chromium content, which helps extend the incubation period.
(2) The porosity of coating mainly influences the corrosion resistance, and the porosity is primarily affected by the oxygen-to-fuel ratio. The coating porosity is also reduced by short and moderate spray distances.
(3) Porosity depends on the thermal energy and kinetic energy of particles. Less porous coatings can be obtained with less thermal energy and high kinetic energy.
(4) The porosity reduces by increasing the oxygen-to-fuel ratio, and decreased porosity is the reason for improved corrosion resistance.
(5) The oxygen flow rate depends on the fuel and spray gun used during the coating process. Likewise, the gas flow rate depends on the coating material used.
(6) The gas flow rate would be comparatively low for material if the size of the particles is fine or if it is more disposed to oxidation than other alloys.
(7) For different materials, different spray distances are used. The standoff distance for a thermal spray process depends on the spray gun used.

## Figures and Tables

**Table 1:** Summary of coating materials and oxygen flow rates used in HVOF.

Fuel	Oxygen Flowrate	Coating Material	Substrate	Remarks	Ref.
LPG	250 LPM	WC-12Co-10-15%Al_2_O_3_	Stainless steel 304	WC-12Co-15Al_2_O_3_ coating showed superior corrosion resistance to WC-12Co-10Al_2_O_3_ coating due to low porosity.	[79]
LPG	250 LPM	WC-Cr_3_C_2_-Ni and WC-17Co	ASME SA213-T22 and ASME SA213-T91	The corrosion resistance of ASME SA213-T91) alloy coated with 93(WC-Cr_3_C_2_)-7Ni was better than other specimens in an actual boiler environment.	[80]
Kerosene	900 LPM	FeCrMnWMoSi amorphous and nanocrystalline	304 stainless steel	The corrosion performance of the coating was good under acidic and neutral media but not suitable under alkaline conditions.	[81]
Kerosene	967 LPM	Fe-based alloy powder	Low carbon steel	The Fe-based coating’s corrosion resistance was higher than that of the electroplated hard chromium coating in HCL and 3.5% NaCl solution due to fewer defects and a dense structure.	[82]
Kerosene	900 LPM	WCCo-Al	Al alloy AA2024	The corrosion resistance of the coating increased as the amount of WCCo increased in the coating layer in corrosion tests.	[83]
LPG	250 LPM	NiCrAlY-SiC and NiCrAlY-B_4_C	T 22 steel	The coating’s hot corrosion behavior was studied at 900 °C in a Na_2_SO_4_-V_2_O_5_ molten salt environment for 50 cycles. NiCrAlY-SiC brought a better corrosion resistance than NiCrAlY-B_4_C coating.	[84]
Kerosene	1900 scfh (893LPM)	Cr_3_C_2_-NiCr	Q 345 steel	The corrosion behavior of the coating was probed using 3.5% wt. NaCl and 3.5 wt.%NaCl with 20 ppm Na_2_S solution for 70 days. The coating revealed an improved corrosion resistance in NaCl with 20 ppm Na_2_S compared to NaCl.	[85]
Kerosene	90 LPM	WC-10Co4Cr with micron-size WC(conventional) and a mixture of nano and micron-sized WC particles (bimodal).	ANSI 4135 steel	The coatings were continuously immersed in drilling fluid simulated seawater at room temperature for 1, 7 and 15 days. The results showed that bimodal coating has a better corrosion resistance and a lower porosity than the conventional coating.	[86]
LPG	250 LPM	93(WC-Cr_3_C_2_)-7Ni, 75Cr_3_C_2_-25NiCr, 83WC-17CO and 86WC-10CO-4Cr	ASME SA213 T91	The coatings were exposed at 900 °C in an actual boiler atmosphere for 10 cycles, including 100 h each. Among all specimens, the 93(WC-Cr_3_C_2_)-7Ni coating showed the maximum corrosion resistance.	[87]
Kerosene	920 SLPM	WC-17Co, WC-10Co-4Cr and Cr_3_C_2_-25NiCr	AISI 1045 steel	The corrosion behavior of the coatings was studied in an alkaline sulfide solution by immersing the specimens in the solution for 18 days. Corrosion rate analysis showed that the Cr_3_C_2_-25NiCr coating exhibited the best resistance against corrosion due to the lowest porosity of 0.87%.	[88]
Propane	240 SLPM	Cr_3_C_2_-25NiCr, Cr_3_C_2_-50NiCrMoNb and Cr_3_C_2_-37WC18NiCoCr	Low carbon steel (grade P92)	The coatings were tested under KCl deposit at two different temperatures of 450 and 550 °C in a furnace for 168 h. The most resistant coating was Cr_3_C_2_-37WC-18NiCoCr at 450 °C.	[89]
LPG	250 LPM	Cr_3_C_2_-25NiCr and WC-10CO-4Cr	ASME SA213 T91, ASME SA213 T22	Both bare steel and coated alloys were exposed to cyclic exposure in a coal-fired boiler at 900 °C for 10 cycles. Both coatings showed a better corrosion resistance on ASME SA213 T22 alloy. In contrast, the corrosion resistance of the 86WC-10CO-4Cr coating was higher than that of the 75Cr_3_C_2_-25NiCr coating on ASME SA213 T22.	[90]
Propylene	278, 265 LPM	Hydroxyapatite (HA) and 80HA-20TiO_2_	Ti-6Al-4V	The electrochemical technique was used to investigate the performance of coatings in natural Hank’s solution in the absence and presence of bovine serum album for 30 days. The polarization studies indicated that 80HA-20TiO_2_ is the only coating with a narrow passive potential region, −0.4 V to 0 V, showing that adding TiO_2_ is beneficial for the stability of HA coating.	[91]
Kerosene	800 LPM for NiAl, 920 LPM for stellie-6	NiAl and Stellite-6	304 stainless steel	The flow rates of oxygen and kerosene were low for NiAl powder due to its lower flowability. Testing was done in a biomass combustor and industrial-scale boiler. The corrosion resistance of stellie-6 coating was excellent in both combustion systems. In contrast, the anti-corrosion performance of NiAl coating was depleted because of the production of the Al_2_O_3_ layer at the substrate/coating interface.	[92]
Hydrogen	O_2_/H_2_ ratio: 0.34, 0.31	WC-12 wt.% Co	Magnesium alloy ZE41	Two O_2_-H_2_ ratio values were used to assess the effect of gas flow transport on the coating morphology. The lowest amount of porosity was gained by the O_2_/H_2_ ratio of 0.34. Due to this low porosity, the coating with two layers protected the substrate from 3 wt.% NaCl solution for 96 h.	[93]
Kerosene	566.4 SLPM	Al_62.5_ Cu_25_Fe_12.5_ and Al_67_Cu_20_Fe_5_Cr_8_	Ferritic stainless steel	The corrosion resistance was measured in acidic and alkaline media in the presence and absence of chlorides. After the electrochemical test, the Al-Cu-Fe-Cr coating performed better, having a less corroded surface in the presence of chlorides.	[94]
Hydrogen	280 LPM	Cr_3_C_2_-WC-NiCoCrMo and Cr_3_C_2_-(25NiCr)	Inconel 625, P91 stainless steel	A hot corrosion test was performed under cyclic conditions in an NaCl-KCl-Na_2_SO_4_ salt environment at 500 °C. As compared to Cr_3_C_2_-(25NiCr), Cr_3_C_2_-WC-NiCoCrMo coating exhibited a superior resistance against corrosion due to low porosity. The thickness of the corrosion scale was one-third for the Cr_3_C_2_-WC-NiCoCrMo coating compared to Cr_3_C_2_-(25NiCr).	[95]
Natural gas	330 LPM	NiCrWMoCuCBFe	316 stainless steel	The corrosion resistance of the coating was evaluated in artificial seawater after 3, 8, 14, and 20 h of cavitation erosion. With the increase in time, the cavitation erosion rate slowed down and the corrosion performance improved.	[96]
Propane	253 LPM	Cr_3_C_2_ 25(Ni-20Cr)	SAE 1020 carbon steel	To determine the corrosion rate, 3.5 wt.% NaCl solution was used at 25 °C. There was more of an impact of cavitation on the kinetics of corrosion than an impact of corrosion on the cavitation resistance of the coating.	[97]
Propane	215, 220, 250 LPM	WC-10Co-4Cr	AISI 410 stainless steel	The temperature of the particles increased during deposition due to a decrease in the oxygen fraction of the gas mixture composition, which contributed to the reduction of porosity. The coating layer with a lower porosity had a better performance in terms of corrosion resistance.	[98]
Kerosene	533 LPM	Fe_48_Mo_14_Cr_15_Y2C15B6	316 SS	The corrosion behavior of the coating was examined under atmospheric pressure (1 atm) and high hydrostatic pressure (80 atm). Concerning 1 atm, the general corrosion rate of the coating increased at 80 atm.	[99]
Kerosene	900 LPM	CoNiCrAlYSi and Al_2_O_3_- YSZ	Inconel 738	A hot corrosion test was performed for 220 h at 880 °C in an electrical tube furnace. The results showed that the central protective oxide was α-Al_2_O_3_ on the coating.	[100]
Kerosene	850 LPM	CoNiCrAlY/YSZ/YSZ-10 wt.%La_2_O_3_	Inconel 738	The results indicated that the topcoat reduced the infiltration of corrosive components during the hot corrosion test at 880 °C for 400 h.	[101]
Kerosene	920 LPM	WC-12%Co	Carbon steel	The coating produced by the nanostructure HVOF procedure had the best microstructure due to a low porosity.	[102]
Hydrogen	14 LPM	Ni-20%Cr and Cr_3_C_2_-25%	A 286 superalloy	The uncoated superalloy severely suffered from hot corrosion under cyclic conditions at 700 °C compared to the coating.	[103]

**Table 2:** Summary of coating materials and spray distances used in HVOF.

Substrate	Coating Material	Spray Distance (mm)	Remarks	Study
Mild steel	Fe_3_Al/TiC and Fe_3_Al-Cr/TiC	380	The corrosion resistance of Fe_3_Al/TiC coating increased during the electrochemical test after adding chromium.	[117]
304 stainless steel	Fe_3_Al	380	Electrochemical impedance spectroscopy revealed that the coating corrosion resistance is inferior because of porosity and cracks.	[118]
Monel K500	WC-10 wt.% Ni-5 wt.% Cr, WC-18 wt.% Hastelloy C	330	HVOF-coated samples showed a better corrosion resistance for 1533 h than that for the uncoated substrate.	[119]
Stainless steel	WC-10Co-4Cr, WC-12Co, Cr_3_C_2_-NiCr	450, 420, 400	WC-10Co-4Cr coating with a low porosity and high toughness showed the best erosion resistance.	[120]
AISI 420 stainless steel	Ni_3_Ti and Ni_3_Ti+(Cr_3_C_2_ + 20NiCr)	200–250	The thermocyclic hot corrosion test was conducted in a molten salt environment for nearly 50 cycles at 650 °C.	[121]
-	WC-12Co and CeO2 modified WC-12Co	350	The coating was tested in 1 mol/L H_2_SO_4_ and 3.5 wt.% NaCl solution for 168 h. The corrosion resistance was better in the NaCl solution than that in the H_2_SO_4_ solution for both CeO2 modified WC-12Co and WC-12Co coatings.	[122]
Grey cast iron	Inconel 718-nano-Al_2_O_3_	200	The hot corrosion performance of coated and bare specimens was evaluated in a high-temperature furnace at 900 °C for 50 h. The composite coating containing 30 wt.% of Al_2_O_3_ content exhibited maximum corrosion resistance and hardness.	[123]
SUS 316	Fe_40_-Cr_19_-Mo_18_-C_15_-B_8_ alloy	330–360	A rotating corrosion tester was used for the corrosion test. The testing duration was 2400 s, and the rotating speed of the spindle was 200 rpm. The coating’s potentiodynamic polarization curves were measured in four solutions: seawater, 0.5 M H_2_SO_4_, 3.5 wt.% NaCl, and 1 M NaOH. The coating revealed an excellent corrosion resistance, with a 185 nm depth in the coating surface.	[124]
AISI 304 stainless steel	Al_2_O_3_-30(Ni20Al)	200	Both the coated and uncoated samples were subjected to thermal cycling tests at 750 and 850 °C in an air atmosphere for 2, 6, 10, and 15 days of exposure time. There was no oxidation on the coated samples, but the uncoated samples were harshly corroded.	[125]
T 22 boiler tube steel	Ni22Cr10Al-1Y and Ni–22Cr–10Al–1Y–20 wt.% SiC	200	The hot corrosion behavior of coated and bare steel was determined in a molten salt environment for 50 cycles at 900 °C. The performance of the composite coating Ni–22Cr–10Al–1Y–20 wt.% SiC, with a low porosity of 0.97%, was better than that of the Ni22Cr10Al-1Y coating, with a porosity of 1.7%.	[126]
AISI 440C stainless steel	WC-12 wt.% Co	380	The corrosion performance of conventional and suspension WC-Co powders was compared by an electrochemical test. Compared to traditional HVOF, the suspension HVOF technique showed a lower corrosion resistance.	[127]
Low carbon martensitic stainless steel.	Cr_3_C_2_-25NiCr, WC-25WB-10Co-5NiCr, WC-10Co-4Cr and MoB-25NiCr	350	Compared to other coatings, the WC-25WB-10Co-5NiCr coating was the most compact, with no micro defects, and it had the highest corrosion resistance. Cr_3_C_2_-25NiCr had the lowest corrosion resistance in a NaCl solution and deionized water.	[128]
316 SS	WC-10Co4Cr	300	The sealed, treated WC-10Co4Cr coating showed a higher corrosion resistance than the sprayed WC-10Co4Cr coating.	[129]
316 SS	CrN film and Cr_3_C_2_-20NiCr interlayer	160	The hot corrosion behaviors of single CrN film, Cr_3_C_2_-20NiCr, and duplex CrN/Cr_3_C_2_-20NiCr coatings were compared at 550 °C for 50 h. The duplex coating exhibited the lowest weight gain after 50 h.	[130]
T24 and T92 steel	NiMoCrW and CoNiCrAlY	250	The coatings were analyzed in a salt mixture of NaCl-KCl and dry air atmosphere for 360 h at 650 °C. Pointedly, the corrosion resistance of both coatings was lower in the salt mixture due to the active oxidation induced by chlorine. However, compared to NiCr coating, these coatings presented a higher corrosion resistance.	[131]
T 24 steel	Ni20Cr	250	The samples were subjected to deposits of KCl, NaCl, K_2_SO_4_, and Na_2_SO_4_ for 360 h in dry air at 650 °C. Sulfate-based salts were not the cause of severe damage; while chloride-based deposits were responsible for aggressive damage. However, the coating maintained the substrate integrity.	[132]
Inconel 718	Co-32Ni-21Cr-8Al-0.8Y and Gd_2_Zr_2_O_7_ (topcoat)	200	A hot corrosion test was conducted in molten salts with 5-, 10-, 15-, and 20-h cycles at 1000 °C. The performance of Gd_2_Zr_2_O_7_ deposited by EB-PVD was superior in hot corrosion compared to that of the conventional YSZ coating system.	[133]
AISI 1045 steel	WC-CoCr	330	HVOF-sprayed coating was sealed with an aluminum phosphate sealing agent, and then the corrosion behavior of the sealed and unsealed coatings was compared in different environments. It was demonstrated that sealant improved the corrosion resistance of the coating.	[134]
304 stainless steels	Ni69Al31	356	A corrosion test was conducted at 700 °C for 250 h. Severe corrosion was observed due to Al growth. It was concluded that direct exposure of O/Cl_2_ gases to the steel/coating interface should be avoided	[135]
AISI 4340 steel	WC-10Co4Cr	300 and 260	The corrosion resistance of a hydrogen-fueled HVOF gun with a standoff distance of 260 mm was slightly higher than that of a kerosene fuel gun with a standoff distance of 300 mm. This happened due to the decarburization of WC particles at higher temperatures and a lower porosity.	[136]
AISI 1045 steel	WC-10Co-4Cr	330	The coating’s microbial-influenced corrosion behavior was studied using ultrasound-assisted sealing of the aluminum phosphate. The ultrasound-assisted sealing enhanced the corrosion resistance of the coating.	[137]
316 L stainless steel	FeCrMnWMoSi	380	The Fe-based coating performed better against pit corrosion in seawater than stainless steel.	[138]
Carbon steel	WC-40Cr_3_C_2_-25NiCr, WC-10Co4Cr and Cr_3_C_2_-25NiCr	380	The corrosion resistances of two powders—except Cr_3_C_2_-25NiCr—were higher than that of the electrolytic hard chrome coating.	[139]
1045 steel substrate	WC-10Co4Cr	420	The results showed that the coating showed a dense microstructure, a low porosity, and no negative transformation in saturated saltwater.	[140]
Stainless steel	WC-10Co-4Cr	300	The coating influenced the corrosion resistance in sea water compared to the steel substrate.	[141]
Inconel 625	NiCoCrAlYTa and YSZ	250	The hot corrosion test was conducted for 52 h at 1000 °C. The oxidation compounds formed can damage the coating deposited by APS more than those using the HVOF technique.	[142]
Low carbon steel	Inconel 718	254	Most oxide contents were iron and chromium. The chromium oxide layer was not stable at high temperatures after the corrosion test.	[143]

**Table 3:** Relation between the coating material, oxygen flowrate, spray distance, and porosity.

Sr No.	Coating Material	Oxygen Flowrate (L/min)	Spray Distance (mm)	Porosity (%)	Ref.
1	CrCNiCr	253	250	0.9	[97]
2	CrCNiCr	935	380	0.9	[139]
3	CrCNiCr	950	400	0.74	[120]
4	FeMoCrYCB	533	350	0.3	[99]
5	PWD1008	355	25.4	1.22	[143]
6	TiCFeCrAl	214	240	0.82	[62]
7	TiCFeCrAl	188	240	0.96	[62]
8	NiCrAlY	250	200	1.64	[84]
9	NiCrAlY	245	210	1.5	[123]
10	NiCrAlYSiC	250	200	0.95	[84]
11	NiCrAlYSiC	250	200	1.57	[84]
12	NiCrAlYSiC	250	200	1.63	[84]
13	WCCoCr	215	200	1.43	[98]
14	WCCoCr	215	230	1.98	[98]
15	WCCoCr	220	200	0.75	[98]
16	WCCoCr	220	230	3.01	[98]
17	WCCoCr	250	230	0.3	[98]
18	WCCoCr	943	330	0.53	[137]
19	WCCoCr	943	370	0.31	[137]
20	WCCoCr	100	240	1.28	[63]
21	WCCoCr	935	380	0.13	[63]
22	WCCoCr	835	350	0.5	[44]
23	WCCoCr	950	450	0.87	[120]
25	In718Al_2_O_3_	270	200	1.2	[123]
26	YSZ	130	250	6.5	[142]
27	Woka400	900	300	0.76	[54]
28	DJ2600	280	260	0.62	[54]
29	WCCo	950	420	0.99	[120]
30	CoNiCrAlY	250	200	2.5	[133]
31	HC	893	300	1.19	[85]
32	Fe alloy	864	280	1.66	[82]
33	Fe alloy	940	280	1.74	[82]
34	Fe alloy	1015	280	1.78	[82]
35	Fe alloy	864	330	0.49	[82]
36	Fe alloy	940	330	1.06	[82]
37	Fe alloy	1015	330	3.19	[82]
38	Fe alloy	864	380	1.42	[82]
39	Fe alloy	940	380	1.76	[82]
40	Fe alloy	1015	380	0.53	[82]
41	Fe alloy	967	330	0.48	[47]
42	HAS	850	380	3.37	[119]
43	WCN	950	300	4.18	[119]
44	CoNiCrAlY/YSZ/La_2_O_3_	850	200	1.5	[118]
45	Al_2_O_3_-NiAl	231	250	2	[125]
46	Stellite6	830	250	1.5	[113]
47	Stellite6	214	250	0.51	[66]
48	Stellite6	201	250	0.89	[66]
49	Stellite6	188	280	4.09	[66]
50	WCCrCNiCr	935	380	0.87	[139]

## Data Availability

Not applicable.
